# Functionalized Gold Nanoparticles as Contrast Agents for Proton and Dual Proton/Fluorine MRI

**DOI:** 10.3390/nano9060879

**Published:** 2019-06-13

**Authors:** Maria Şologan, Francesco Padelli, Isabella Giachetti, Domenico Aquino, Mariangela Boccalon, Gianpiero Adami, Paolo Pengo, Lucia Pasquato

**Affiliations:** 1 Department of Chemical and Pharmaceutical Sciences, University of Trieste, Via L. Giorgieri 1, 34127 Trieste, Italy; Mariangela.Boccalon@bracco.com (M.B.); gadami@units.it (G.A.); ppengo@units.it (P.P.); 2 Neuroradiology Unit, Fondazione IRCCS Istituto Neurologico Carlo Besta, 20133 Milano, Italy; isabella.giachetti@istituto-besta.it (I.G.); Domenico.Aquino@istituto-besta.it (D.A.); 3 INSTM Trieste Research Unit, Via L. Giorgieri 1, 34127 Trieste, Italy

**Keywords:** hybrid organic-inorganic nanoparticles, magnetic resonance imaging, gadolinium, contrast agents, fluorinated monolayers, fluorine, ^19^F MRI

## Abstract

Gold nanoparticles carrying fluorinated ligands in their monolayer are, by themselves, contrast agents for ^19^F magnetic resonance imaging displaying high sensitivity because of the high density of fluorine nuclei achievable by grafting suitable ligands on the gold core surface. Functionalization of these nanoparticles with Gd(III) chelates allows adding a further functional activity to these systems, developing materials also acting as contrast agents for proton magnetic resonance imaging. These dual mode contrast agents may allow capitalizing on the benefits of ^1^H and ^19^F magnetic resonance imaging in a single diagnostic session. In this work, we describe a proof of principle of this approach by studying these nanoparticles in a high field preclinical scanner. The Gd(III) centers within the nanoparticles monolayer shorten considerably the ^19^F T1 of the ligands but, nevertheless, these systems display strong and sharp NMR signals which allow recording good quality ^19^F MRI phantom images at nanoparticle concentration of 20 mg/mL after proper adjustment of the imaging sequence. The Gd(III) centers also influence the T1 relaxation time of the water protons and high quality ^1^H MRI images could be obtained. Gold nanoparticles protected by hydrogenated ligands and decorated with Gd(III) chelates are reported for comparison as ^1^H MRI contrast agents.

## 1. Introduction

Gold nanoparticles (AuNPs) are ideal scaffolds to set up prototypes for therapy and diagnosis due to the variety of experimental synthetic procedures to control their size, shape, and dispersion. Indeed, the properties of the gold core and shell can be exploited for imaging as well as for therapy. Additionally, the organic coating that stabilizes the metal surface can be conveniently tailored to control the interaction of the NPs with the biological system [1,2,3] and to introduce recognition elements and/or specific units for additional imaging [4]. A diagnostic procedure that would benefit from the use of nanostructured materials and AuNPs in particular is magnetic resonance imaging (MRI).

**Scheme 1:**
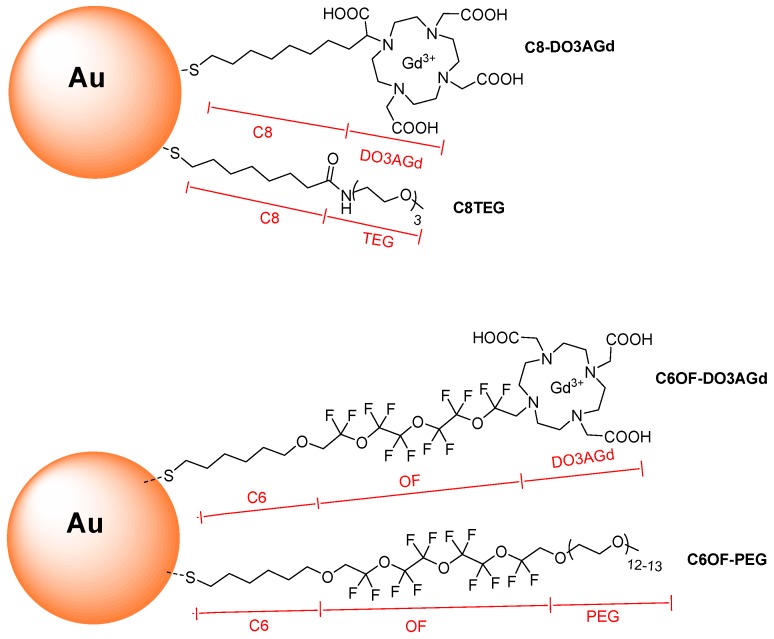
Structure of thiolated ligands used in this study to prepare gadolinium-functionalized nanoparticles.

The conventional proton MRI techniques exploit the NMR signals arising from the hydrogen nuclei of the water molecules within the tissue under analysis and require the use of contrast agents (CA) to provide the necessary contrast for obtaining detailed images of organs and tissues in vivo [5]. The CA can act either on the T1 or T2 relaxation times of the water protons, the former are widely used in the clinical practice and largely based on Gd(III) complexes, the latter instead are mainly based on superparamagnetic iron oxide nanoparticles (SPIONs) [6]. The performance of a CA is determined by its relaxivity, which defines the dependence of relaxation rates R1 or R2, defined as 1/T1 or 1/T2 respectively, on the concentration of the contrast agent. Relaxivity has to be maximized when aiming at developing high performance CA. As far as gadolinium complexes are concerned, the most important parameters affecting relaxivity are the hydration number of the complex, the water exchange rate constant, the rotational correlation time of the complex and the electron relaxation parameters [7]. A first strategy to improve the relaxivity could be increasing the hydration number; however, this generally affects negatively the stability of the complex which may result in displacement of the water molecules with endogenous anions, like carbonate or phosphate [8] or transmetallation with release of toxic Gd(III) ions [9]. A viable approach to develop gadolinium-based contrast agents with improved performance is to increase relaxivity by slowing the molecular tumbling rate. Indeed, the tumbling rate directly affects the T1 relaxation time as can be inferred from the Solomon–Bloembergen–Morgan Equations [10]. This has been achieved by conjugating low molecular weight CA with organic macromolecules, or core-shell inorganic nanoparticles [11,12] and monolayer protected AuNPs. With reference to the latter, Meade and coworkers reported Gd-decorated AuNPs displaying relaxivities per gadolinium center ranging from 13.7 to 14.6 mM^−1^ s^−1^ at 1.41 T which drop to 4 and 4.7 mM^−1^ s^−1^ respectively at 7 T [13]. As a reference value, the widely used CA Dotarem (Gd-DOTA), has relaxivities of 4.6 mM^−1^ s^−1^ at 1.5 T and 4.2 mM^−1^ s^−1^ at 7 T in whole blood at 37 °C [14]. Helm reported an impressive 60 mM^−1^ s^−1^ (30 MHz, 0.71 T at 25 °C) relaxivity per Gd(III) chelate unit immobilized on AuNPs using Gd(III) chelates derived from diethylenetriaminetetraacetic acid (DTTA)-type ligands [15]. By contrast, Workentin and Hudson reported water soluble AuNPs decorated with triethylene glycol terminated with maleimide conjugated to over 50 Gd(III) chelators prepared via an interfacial Michael addition in aqueous media [16]. In this nanoparticle system, the relaxivity per gadolinium unit was the same scored by the monomeric gadolinium complex: about 2 mM^−1^ s^−1^ at 9.4 T. In an interesting work by Penadés and coworkers, describing the preparation of sugar/Gd-coated AuNPs for cell labeling and tracking by MRI, the authors point out that higher reflexivity values can be achieved for nanoparticle systems in which the gadolinium complex are tightly packed within the monolayer [17]. The fact that relaxivity values, besides the size of the scaffold, are affected by the local environment of the gadolinium centers has been pinpointed by Meade and coworkers [18] who provided evidence that particle scaffolds with irregular shape such as gold nanostars provide gadolinium-based nanostructured CA with extremely high relaxivities, up to 98 mM^−1^ s^−1^ per gadolinium unit, at 32 MHz corresponding to a magnetic field of 0.75 T. In addition, further studies from the same group revealed that the increased number of nanostar branches was correlated with enhanced relaxivity [19]. Even though proton MRI is well-established, ^19^F MRI presents advantages with respect to the former; ^19^F MRI scarcely shows background signals in animal bodies and allows quantitative determinations [20,21], in addition ^19^F is a highly sensitive NMR nucleus with 100% natural abundance and receptivity close to that of protons [22]. Moreover, since ^1^H and ^19^F have very similar gyromagnetic ratio ^19^F MRI can be potentially run on existing ^1^H imaging hardware, allowing dual imaging in a single diagnostic session, as already available in some clinical instrumentation. ^19^F MRI requires the use of fluorinated contrast agents to provide the MRI signal, in most cases micelles, dendrimers [23], and nanoemulsions [24,25,26,27]. In our group, water soluble AuNPs functionalized with a fluorinated polyethylene glycol derivative with features suitable for ^19^F-MRI were recently reported [28]. Our studies have so far demonstrated that fluorinated AuNPs are compatible with the biological environment and are well tolerated by model cell lines [29]. Furthermore, the high affinity of small drug-like molecules for fluorinated monolayers may represent a tool for developing theranostic devices which couple their potential drug delivery ability [30] with diagnostic features [28,31]. However, fluorinated compounds can have relatively long relaxation times, resulting in long acquisition times [32]. This problem can be circumvented by introducing in the structure of the ^19^F CA paramagnetic centers, such as Gd(III) in the proximity of ^19^F nuclei, shortening T1 and allowing for the acquisition of a larger number of scans in the same experimental time [33,34,35,36]. In this context, Yu and coworkers reported that in a small, water soluble compound having nine equivalent fluorine atoms which is able to chelate gadolinium ions [37] the T1 shortening enabled by the paramagnetic center significantly improved the signal to noise ratio. A similar effect was observed in AuNPs decorated, via hydrogen bonds, with fluorinated guanidinium amphiphiles and carrying in their monolayer also thiolates capable of complexing Gd(III). In this system a 27% decrease in ^19^F T1 could be achieved [33]. However, the stability and compatibility of these NPs, obtained from phosphine-stabilized gold clusters of 1.5 nm in diameter, with biological media and their toxicity with cells were not investigated. Based on these evidences, we explored the introduction of Gd(III) complexes in the monolayer of fluorinated AuNPs, aiming at decreasing the T1 and T2 relaxation times of proton and fluorine and to assess the viability of these systems in dual ^1^H/^19^F MRI using a preclinical setup. For this purpose, the two classes of mixed monolayer AuNPs with a gold core size of 1.5–2.0 nm, represented in Scheme 1, were designed and synthesized.

The first type of nanoparticles is protected by a mixture of the thiolate **C8TEG**, which is the deprotonated form of *N*-1-{2-[2-(2-Methoxyethoxy)ethoxy]ethyl}-8-sulfanyloctanamide (**HS-C8-TEG**), and a new ligand presenting an eight carbon atom chain ending with the tetraaza macrocycle for gadolinium complexation, named **C8-DO3AGd**. The gadolinium chelating unit of this ligand is based on the 1,4,7,10-tetraazacyclododecane-1,4,7,10-tetraacetic acid (DOTA) scaffold, the synthesis and characterization of this ligand is reported in the Supplementary material (SM). For the synthesis of the second class of nanoparticles we used a previously reported fluorinated thiol **HS-C6OF-PEG** [28] and a functionalized derivative of this ligand which can bind the gadolinium ion **HS-C6OF-DO3AGd**.

## 2. Materials and Methods

### 2.1. General Information

All commercially available reagents were purchased from Aldrich (Milan, Italy) and Alfa Aesar (Karlsruhe, Germany), with exception of tri-tert-butyl-2,2′,2″-(1,4,7,10-tetraazacyclododecane-1,4,7-triyl)triacetate (DO3AtBu), which is a product of CheMatech (Dijon, France). Reagents were used without purification unless otherwise mentioned. Gold and gadolinium standard solutions for ICP–OES analysis were obtained from Sigma–Aldrich (Milan, Italy). Solvents were obtained from Aldrich and VWR, deuterated solvents from Cambridge Isotope Laboratories and Aldrich. Dry solvents were obtained from Aldrich and Alfa Aesar. Chlorinated solvents were kept over K_2_CO_3_ with occasional shaking for at least 24 h prior to use. All other solvents were reagent grade and used as received. Purifications by flash chromatography were performed on Merck silica gel 60F-254 (230–400 mesh). Routine nuclear magnetic resonance analyses were performed on a Varian 500 spectrometer operating at 500 MHz for ^1^H, 470 MHz for ^19^F and 125 MHz for ^13^C.

### 2.2. Synthesis of **NP-C8TEG/C8-DO3AGd**

**NP-C8TEG/C8-DO3AGd-a**: 27.4 mg of **NP-C8TEG** (synthesized as described in the Supplementary material) were dissolved in 12 mL of deoxygenated mQ water and the solution was added at the **HS-C8-DO3AGd** in HEPES buffer using a ratio of 1.3/1 between the Gd-chelating ligand and the ligands grafted on the nanoparticle surface. The reaction mixture was allowed to stir at 25 °C for 3 days. After this time, the nanoparticles were transferred in one Amicon centrifuge filter (10000 MWCO). Then the nanoparticles were washed with milliQ water (7 × 20 mL) and after that they were dried giving a brown solid. ICP-OES: [Au]/[Gd] = 13; 17 moles of Gd/NPs. TGA: 41.4%; Au_220_C8TEG_63_/C8-DO3AGd_17_.

**NP-C8TEG/C8-DO3AGd-b**: A solution containing 17.5 mg of **NP-C8TEG** in 10 mL of deoxygenated milliQ water were added to 1 mL of 0.1 M HEPES buffer, pH 7.4, containing **HS-C8-DO3AGd** (2.1 Equiv. respect to the nanoparticle ligands) and the solution was allowed to stir at 40 °C for 3 days. Then the nanoparticles were purified following the same protocol as described for **NP-C8TEG/C8-DO3AGd-a** obtaining analytically clean nanoparticles. An aliquot of 1 mg of these nanoparticles was digested with aqua regia for the ICP-OES analysis, obtaining an [Au]/[Gd] ratio of 8.3. TGA: 43.6%. The average composition of **NP-C8TEG/C8-DO3AGd-b** was determined as Au_220_C8TEG_53_C8-DO3AGd_27_.

### 2.3. Synthesis of **NP-C6OF-PEG**

A solution of tetrachloroauric acid (0.058 g, 0.1465 mmol) was dissolved in 50 mL of deoxygenated water and the thiol **HS-C6OF-PEG** (0.310 g, 0.293 mmol) dissolved in 50 mL deoxygenated methanol was added to the solution. The mixture was stirred for 30 min at room temperature and for 30 min at 0 °C and then a freshly prepared solution of NaBH_4_ (0.061 g, 1.6115 mmol) was rapidly added. The reaction mixture was stirred for 1 h at 0 °C and 2 h at room temperature, the solvent was removed without exceedingly concentrating the solution and milliQ water was added at small portions. The resulting solution was dialyzed for 3 days against milliQ water. Removal of the solvent under reduced pressure left a brown solid that was further purified washing with diethyl ether (2 × 15 mL). The solvent was removed obtaining 0.080 g of **AuNPs-C6OF-PEG**. ^1^H-NMR (CD_3_OD, 400 MHz) δ: 3.38 (br, CH_3_O), 3.48–3.77 (br, CH_2_O), 4.10 (br, CF_2_CH_2_O). UV-VIS λ_max_: 540 nm (weak). TEM: *D* = 1.5 nm; σ = 0.3 nm. TGA: 68.5%. Hydrodynamic diameter (water): 15 ± 12 nm. TGA: 68.5%. Average composition: Au_130_C6OFPEG_53_.

### 2.4. Synthesis of **NP-C6OF-PEG/C6OF-DO3AGd**

**NP-C6OF-PEG** (25 mg) was dissolved in 2 mL of deoxygenated milliQ water and the mixture was added to 2 ml of 0.1 M HEPES buffer pH 7.4 containing 0.032 mmol of **HS-C6OF-DO3AGd** corresponding to a ligand ratio of 2/1 between the incoming ligand and the ligands on the nanoparticles monolayer; the reaction mixture was stirred at 40 °C for 3 days. After this time, the nanoparticle solution was transferred into an Amicon centrifugal filtration device (10000 MWCO) and centrifuged at 4500 rpm for 30 min, then the nanoparticles were washed with milliQ water (7 × 20 mL). After removal of the solvent, 20 mg of nanoparticles were obtained as a brown solid. ICP-OES: [Au]/[Gd] = 11.55; TGA: 73%. Au_130_C6OFPEG_53_C6OF-DO3AGd_12_. TEM: *D* = 1.5 nm, σ = 0.3 nm.

### 2.5. Determination of the Au/Gd Ratio

Total Au and Gd concentrations in the samples, solubilized with 1 mL of aqua regia (mixture HCl:HNO_3_ of 3:1) and diluted to 10 mL with MilliQ water, were quantified by means of inductively coupled plasma-optical emission spectrometry (ICP-OES) using an Optima 8000 instrument (PerkinElmer; Waltham, MA, USA) equipped with an S10 integrated autosampler (PerkinElmer; Waltham, MA, USA). The measurements were conducted using a calibration curve obtained by dilution of gold and gadolinium standard solutions for ICP–OES. The limit of detection in the obtained solutions at the operative wavelengths (Au 267.595 nm and Gd 376.839 nm) was 0.02 mg L^−1^ for both Au and Gd. The precision of the measurements expressed as repeatability (as RSD %) for the analysis was always less than 5%.

### 2.6. Determination of Nanoparticles’ ^19^F Relaxation Times

Relaxation times T1 and T2 for the fluorinated nanoparticles were determined at 470 MHz using a Varian 500 MHz NMR spectrometer and the VNMRJ software version 3.1. Optimization of the 90° pulse was carried out before determining the longitudinal and transverse relaxation times for all samples. Assessment of T1 was performed by inversion recovery using the pulse sequence INVREC with a relaxation delay of 2.4 s, and 16 scans per experiment, 11 experiments. Determination of T2 was performed using the Carr-Purcell-Meiboom-Gill pulse sequence, CPMGT2 with a relaxation delay of 0.02 s, and 16 scans per experiment, 11 experiments. The samples were prepared by dissolving 19 mg of nanoparticles in 0.8 mL of deuterated solvents.

### 2.7. Determination of T1 and T2 Values with the 7 T Preclinical Scanner

#### 2.7.1. ^1^H Experiments

Magnetic resonance imaging (MRI) and spectroscopy (MRS) experiments were performed by a horizontal-bore preclinical scanner (BioSpec 70/20 USR, Bruker, Ettlingen, Germany). The system has a magnetic field strength of 7 T and a 20 cm bore diameter. The scanner is equipped with an actively shielded gradient system with integrated shims set up to 2nd order. The maximum gradient amplitude is 440 mT/m. Acquisitions were carried out using a transceiver double tunable ^1^H/^19^F linear birdcage RF coil having a diameter of 72 mm. All measurements were done at room temperature. Test tubes were placed at the gradient isocenter and the magnetic field homogeneity within the field of view (FOV) was guaranteed by a 1st order shimming procedure.

^1^H relaxometry was performed on test tubes containing solutions of functionalized nanoparticles with concentrations of 40; 20; 10 mg/mL. Estimation of T1 and T2 relaxation times was obtained by means of a set of rapid acquisition with relaxation enhancement (RARE) sequences varying repetition time (TR) and echo time (TE), respectively, see SI for further details. For each sample ^1^H MRI were obtained by RARE sequences whose parameters were set up according to the previously determined T1 and T2 values.

#### 2.7.2. ^19^F Experiments

For **NP-C6OF-PEG** and **NP-C6OF-PEG/C6OF-DO3AGd**, the mother solutions’ nonlocalized ^19^F MRS data were acquired by means of a single pulse excitation sequence (TR = 2000 ms, Flip Angle = 90°, excitation bandwidth = 14 kHz, receiver bandwidth = 100 kHz, spectral resolution 12.21 Hz, 2000 averages for 33 min of acquisition time), setting the correct excitation frequency, optimizing the excitation bandwidth and the pulse gain.

The protocol for ^19^F MRI was optimized to obtain the best sensitivity from the ^19^F signal. The MR examination of the samples was carried out by means of a RARE sequences varying TR, TE, RARE factor (equivalently to the echo train length (ETL), it represents the number of echoes registered in one TR), slice thickness, and matrix size. The resulting optimal setting was used in the subsequent acquisitions. In order to evaluate the minimal concentration detectable in an acquisition time compatible with future in vivo applications, the same protocol was applied to the mother solution (40 mg/mL) and to the diluted samples (20 mg/mL).

### 2.8. Dynamic Light Scattering

Dynamic light scattering (DLS) measurements were performed on a Malvern Zetasizer Nano (Malvern, UK) in backscatter mode. Analyses were performed on nanoparticle solutions with concentration of 10 mg/mL or 3.7 mg/mL, using either disposable microcuvettes or disposable zeta cells (Malvern). The cell positioning factor was set to 0.83 mm and attenuator values of five or nine were auto-optimized as a function of nanoparticle concentration.

## 3. Results

AuNPs are particularly suited as a scaffold to develop nanostructured gadolinium-based MRI contrast agents with improved performance respect to monomeric gadolinium complexes. Indeed, with respect to other high molecular weight species, the control of their size (and thus the effect on the longitudinal relaxation time) can be easily achieved during the nanoparticles’ preparation. Furthermore, AuNPs can be easily functionalized by place exchange reaction under mild conditions, enabling control over the number of gadolinium chelate units in their monolayer by simply acting on the ratio between the bound thiolates and the incoming functionalized ligands. In addition, the ease of functionalization of these systems may even allow increasing the selectivity towards specific cells by installing recognition moieties targeting overexpressed receptors in their monolayer [38]. Moreover, water-soluble AuNPs displaying polyethylene glycol units at their external surface are compatible with the biological environment, display limited toxicity, and may limit the nonspecific adsorption of biomolecules. This is a prerequisite for the development of efficient CA for magnetic resonance imaging, the suitability for this application is also supported by the high concentrations of nanoparticles that can be achieved given the excellent solubility properties ensured by the presence of the PEG layer [39]. Furthermore, it is anticipated that, based on similar PEGylated systems, these should display reduced clearance rate of the nanoparticles after administration [40]. With this in mind, we designed the two nanoparticle systems reported in Scheme 1. In both cases, the nanoparticles feature amphiphilic ligands in their monolayer terminating with a PEG moiety. On these systems we introduced the Gd(III)-chelating ligands which differ in two aspects: the nature of the linker and the type of the chelator. The syntheses and the complete characterization of these ligands are reported in the SM. The first type of AuNPs displays fully hydrogenated capping ligands; in this system, the nanoparticles serve solely as scaffolds for grafting gadolinium chelates on their surface, achieving T1 contrast agents acting on the mobile water molecules in the neighborhood of the nanoparticle surface. Moreover, these AuNPs will serve as reference for the second type of AuNPs. These are characterized by the inner part of the monolayer featuring a fluorinated region in which there are eight quasi-equivalent ^19^F nuclei per ligand [28], and a limited number (12) of ligands to chelate Gd ions. The inner part of the monolayer can be exploited to generate the ^19^F MRI signal while the gadolinium chelates have a two-fold role: on one hand as T1 contrast agents for conventional proton MRI, and on the other hand they help in shortening the T1 of the fluorine nuclei signal as a way to increase the performance of the nanoparticles as ^19^F CA. Overall, this system represents a first step in developing AuNPs-based CA for dual ^1^H and ^19^F MRI.

**Figure 1:**
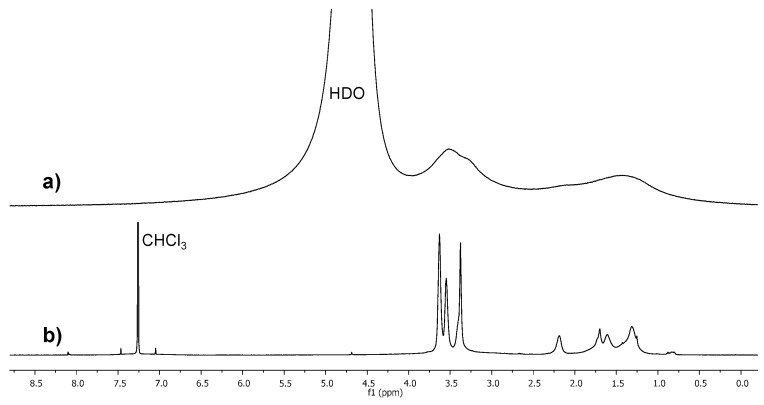
^1^H NMR spectra of (**a**) **NP-C8TEG/C8-DO3AGd-a** in D_2_O (500 MHz) and (**b**) **NP-C8TEG** in CDCl_3_ at 500 MHz.

In order to ensure high stability for the NPs and for the gadolinium complex, the ligands were designed on the DOTA scaffold, which was selected on the basis of its high overall complexing constant for Gd(III), LogK ~ 24 [41], this required the use of the commercially available DO3A building block, which has a LogK ~ 21 [41]. AuNPs protected by the **C8TEG** ligand were synthesized according to a reported procedure developed by some of us in a homogenous phase synthesis using a mixture of methanol/water 1:1 [39] and in order to obtain nanoparticles with a core diameter of about 1.6 nm, we have used an Au/thiol molar ratio of 1/3 ratio. Consistently, the UV-Vis absorption spectrum of the NPs did not display the surface plasmon band at around 520 nm, qualitatively confirming that the nanoparticles are smaller than 2.5–3.0 nm. Indeed, the size of the gold core, determined by TEM analysis, was 1.7 ± 0.3 nm. Combining this result with thermogravimetric analysis (TGA), the average composition was determined as Au_220_C8TEG_63_. The ligands to bind gadolinium ions were introduced in the monolayer of these NPs by ligand exchange reaction using a ratio of 1.3/1 or 2.1/1 between the ligand **C8-DO3AGd** and the **C8TEG** ligands in the monolayer of **NP-C8TEG**; the reactions were performed in 0.1 M HEPES buffer, pH 7.4. The complexation of Gd(III) with the chelating ligand was performed immediately before the place exchange reaction. Two protocols for the place exchange reactions were used, allowing us to prepare AuNPs with two different loadings of gadolinium chelates in order to assess the influence of the amount of the paramagnetic ion on the CA performance. These AuNP are dubbed **NP-C8TEG/C8-DO3AGd-a** and **NP-C8TEG/C8-DO3AGd-b**; the number of Gd chelates introduced into the monolayer of the nanoparticles was determined by ICP-OES and was found to be 17 and 27 chelates/nanoparticle, corresponding to compositions of Au_220_C8TEG_63_C8-DO3AGd_17_ and Au_220_C8TEG_53_C8DO3AGd_27_, respectively. The ^1^H NMR spectrum (Figure 1, trace (a)) of these nanoparticles shows clearly the effect exerted by the presence of the paramagnetic ion, which causes the already broad signals of the nanoparticle **C8TEG** (Figure 1, trace (b) ) to be so severely broadened to be almost indistinguishable from the baseline.

The effect exerted by these NPs on the T1 and T2 relaxation times of the water protons have been initially determined in a 7 T preclinical scanner using aqueous solutions of NPs at a concentration of 40 mg/mL. For **NP-C8TEG/C8-DO3AGd-a**, this corresponds to a 9.2 mM concentration of Gd(III), while for **NP-C8TEG/C8-DO3AGd-b**, this corresponds to a 14.04 mM concentration of Gd(III). The T1 and T2 data obtained for these systems are reported in Table 1.

In the same set-up, the T1 and T2 values of bulk water were determined as 2724 ± 19 ms and 972 ± 6 ms. Therefore, at these concentration levels, **NP-C8TEG/C8-DO3AGd-a** cause a 80-fold reduction in T1 and a 150-fold reduction in T2 of the water protons. In the case of **NP-C8TEG/C8-DO3AGd-b**, the T1 and T2 values were reduced 190-fold and 300-fold respectively. These data are consistent with the different loadings of Gd(III) in these functionalized AuNPs, 17 Gd(III) ions for **NP-C8TEG/C8-DO3AGd-a** and 27 for **NP-C8TEG/C8-DO3AGd-b**. To gain a deeper insight on the intrinsic relaxivities of these systems, a series of experiments were performed by testing **NP-C8TEG/C8-DO3AGd-a** and **NP-C8TEG/C8-DO3AGd-b** at different concentrations, namely 40, 20, and 10 mg/mL. For **NP-C8TEG/C8-DO3AGd-a**, we obtained a r1 relaxivity value of 3.2 mM^−1^ s^−1^, while for **NP-C8TEG/C8-DO3AGd-b**, 4.9 mM^−1^ s^−1^ was obtained (Table 1). Therefore, even though the nanoparticle scaffold in the two cases is the same, the relaxivity value per gadolinium unit is slightly higher in the case of **NP-C8TEG/C8-DO3AGd-b**. This can be accounted for by considering that in the latter case, the nanoparticle monolayer is reasonably more rigid than that of **NP-C8TEG/C8-DO3AGd-a** because of the higher number of gadolinium chelates. This effect is in keeping with the observation of Penadés [17]. Overall, the relaxivities of these AuNPs are in agreement with the values determined at high field for similar gadolinium complexes grafted on AuNPs with gold cores of 1.7–1.8 nm in diameter [17].

**Figure 2:**
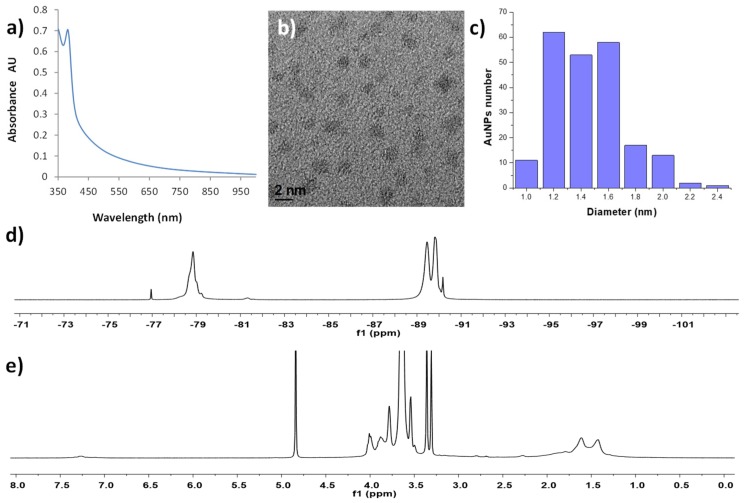
Characterization of **NP-C6OF-PEG**. Panel (**a**) UV-Vis spectrum in CHCl_3_ of the nanoparticles; panels (**b**,**c**) representative TEM image and histogram of the size dispersion; panels (**d**,**e**) ^19^F NMR (CD_3_OD, 470 MHz) and ^1^H NMR (CD_3_OD, 500 MHz) spectra, respectively.

The preparation of AuNPs featuring both a fluorinated monolayer and gadolinium centers has been pursued using the same approach used for **NP-C8TEG/C8-DO3AGd**. In particular, the nanoparticles **NP-C6OF-PEG** were prepared as previously described [28] using a single-phase water/methanol synthetic process and employing a 2:1 thiol to gold ratio. The absence of any surface plasmon band in the UV-Vis spectrum of these species (Figure 2a) qualitatively suggests the formation of AuNPs with a core diameter smaller than 2.5–3.0 nm. Indeed, the TEM analysis (Figure 2b,c) revealed an average core diameter of 1.5 nm with a standard deviation of 0.3 nm (217 measurements). The ^19^F NMR and ^1^H spectra of these AuNPs are reported in Figure 2d,e, respectively.

The ^19^F NMR spectra of these AuNPs display two signals centered at about −90.1 and −79.0 ppm; the former is assigned to the central fluorine nuclei, while the latter is assigned to the terminal fluorine nuclei of the perfluoropolyether moiety of the ligands.

**Figure 3:**
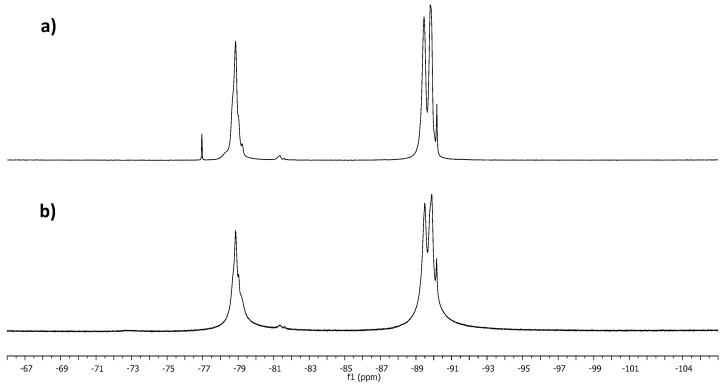
(**a**) ^19^F NMR (470 MHz, CD_3_OD) of NP-C6OF-PEG and (**b**) of NP-C6OF-PEG/C6OF-DO3AGd.

The gadolinium-chelating ligand **HS-C6OF-DO3A** for the functionalization of these nanoparticles was designed to feature the same fluorinated moiety as that of the PEGylated ligands in order to maintain a high number of fluorine nuclei in the monolayer of the nanoparticles. In this case, however, the availability of the fluorinated building block prevented the introduction of a further carboxylic group to complete the coordination sphere of the Gd(III); the synthesis and characterization of the ligand is described in the SM. The complexation of Gd(III) with the ligand was performed immediately before the place exchange reaction with **NP-C6OF-PEG**. The place exchange was carried out using a 2/1 ratio between the bound and the incoming ligand at 40 °C in HEPES buffer for three days. The nanoparticles could be purified by filtration using centrifugal devices with a molecular weight cutoff of 10,000 Da. The **NP-C6OF-PEG/C6OF-DO3AGd** proved to be analytically clean by means of ^1^H and ^19^F NMR spectra. In Figure 3, the ^19^F NMR spectrum of the functionalized nanoparticles is reported in comparison to that of **NP-C6OF-PEG**. As in the case of **NP-C8TEG/C8-DO3AGd**, a clear line broadening in the NMR signals is evident.

Preliminary characterization of the Gd-functionalized NPs in terms of the ^19^F T1 and T2 relaxation times was performed at 470 MHz on a CD_3_OD solution of the nanoparticles considering the signal at −90.1 ppm. The obtained T1 and T2 relaxation times are reported in Table 2. For comparison purpose, the T1 and T2 values were determined also for the uncomplexed **HS-C8OF-DO3A** ligand.

The comparison of the T1 and T2 values of the functionalized and unfunctionalized nanoparticles shows that the introduction of the gadolinium complexes in the monolayer of **NP-C6OF-PEG/C6OF-DO3AGd** causes the expected decrease in ^19^F T1, which is reduced to 36% of that determined for **NP-C6OF-PEG**. This effect was also confirmed by running the T1 determination in the 7 T preclinical scanner on aqueous solutions of both **NP-C6OF-PEG/C6OF-DO3AGd** and **NP-C6OF-PEG**; in this case, the T1 value was found to be reduced to 57% of its original value (Table 2); the exceedingly long T2 prevented its accurate determination in this setup.

**Figure 4:**
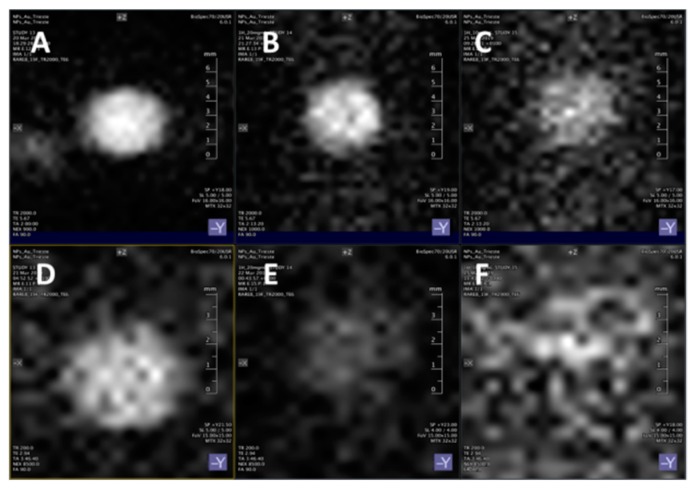
^19^F MRI Phantom experiments run on solutions of: upper panels, **NP-C6OF-PEG** at (**A**): 40 mg/mL; (**B**): 20 mg/mL; (C): 10 mg/mL; lower panels, **NP-C6OF-PEG/C6OF-DO3AGd** at (**D**): 40 mg/mL; (**E**): 20 mg/mL; (**F**): 10 mg/mL.

In the same preclinical setup, both **NP-C6OF-PEG** and **NP-C6OF-PEG/C6OF-DO3AGd** samples displayed high and sufficiently sharp MRS peaks allowing optimization of the excitation frequency and sequence parameters for ^19^F MRI acquisitions. Accordingly, ^19^F MRI phantom experiments were run on nanoparticle solutions with concentrations of 40, 20, and 10 mg/mL. **NP-C6OF-PEG** solutions displayed an NMR signal which remained detectable for all of the concentrations considered (Figure 4A–C). **NP-C6OF-PEG/C6OF-DO3AGd**, although displaying an overall less intense signal, achieved an acceptable signal to noise ratio for both the 40 mg/mL (Figure 4D) and 20 mg/mL concentrations (Figure 4E). Having established the viability of this nanoparticle system as a ^19^F CA, we assessed its ability to shorten the T1 and T2 relaxation times of the water protons using the same preclinical setup. To this end, the same series of nanoparticle solutions with concentrations of 40, 20, and 10 mg/mL, corresponding to concentrations of Gd-chelate units of 5.12, 2.56, 1.28 mM, respectively, was subjected to ^1^H relaxometry experiments that enabled calculating the r1 and r2 relaxivities per gadolinium center. The r1 and r2 relaxivities thus determined were 0.45 mM^−1^ s^−1^ and 4.2 mM^−1^ s^−1^, respectively. Quite interestingly, these values are much smaller than those calculated for **NP-C8TEG/C8-DO3AGd**, indicating that the effect of the paramagnetic centers on the relaxation times of bulk water molecules is less pronounced for **NP-C6OF-PEG/C6OF-DO3AGd**. This effect calls for some considerations: firstly, it is worth pointing out that the structure of the **C6OF-DO3AGd** ligand, which lacks a carboxylic group compared to the ligand **C8-DO3AGd** used in the functionalization of **NP-C8TEG**, see Scheme 1, should have enabled increasing the hydration number of the complex. This possibly increased the relaxivity; however, increasing the number of water molecules bound to the Gd(III) centers is only effective if these water molecules can efficiently exchange with the bulk of the solvent. This seems to be difficult for **NP-C6OF-PEG/C6OF-DO3AGd**, pinpointing either a poor degree of hydration of the region of the monolayer in which the Gd(III) chelates are embedded or a slow transport of water molecules from this region to the bulk. Overall, this suggests that the region at the boundary between the PEG and the oxyfluorinated regions of the monolayer in **NP-C6OF-PEG/C6OF-DO3AGd** is rather hydrophobic as already noted by electron spin resonance (ESR) investigation of the complexation of hydrogenated and fluorinated radical probes for the parent **NP-C6OF-PEG** [30].

**Figure 5:**
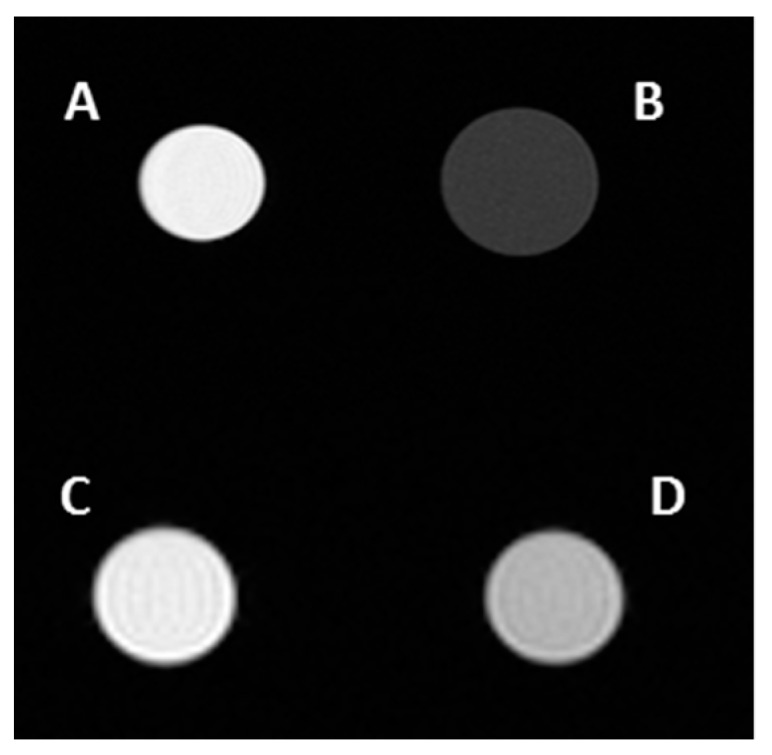
7 T ^1^H MRI images obtained in phantom experiments on solutions of nanoparticles **NP-C6OF-PEG/C6OF-DO3AGd** (**A**); **NP-C6OF-PEG** (**B**); **NP-C8TEG/C8-DO3AGd-a** (**C**); **NP-C8TEG/C8-DO3AGd-b** (**D**). All experiments were performed on 40 mg/mL solutions of the nanoparticles.

Despite the limited effect of the Gd(III) centers embedded in the monolayer of **NP-C6OF-PEG/C6OF-DO3AGd** for the water protons, it was possible to optimize ^1^H MRI acquisition which gave, in phantom experiments, high quality images comparable to those obtained by using solutions of **NP-C8TEG/C8-DO3AGd-a** and **NP-C8TEG/C8-DO3AGd-b**. Figure 5A reports the image obtained in the ^1^H MRI phantom experiment pertinent to a 40 mg/mL solution of **NP-C6OF-PEG/C6OF-DO3AGd** compared to that of **NP-C6OF-PEG**, at the same concentration, used as the reference in Figure 5B. Figure 5C reports the ^1^H MRI for **NP-C8TEG/C8-DO3AGd-a** and Figure 5D reports the phantom experiment for **NP-C8TEG/C8-DO3AGd-b**.

## 4. Conclusions

In this work, we explored the suitability of water-soluble gold nanoparticles protected by a fluorinated organic monolayer carrying Gd(III) complexes as a contrast agent for dual ^1^H/^19^F MRI procedures. This nanoparticle system was compared to both hydrogenated gold nanoparticles decorated with Gd(III) chelates and fluorinated nanoparticles devoid of paramagnetic centers. Embedding Gd(III) in the monolayer of fluorinated nanoparticles reduces the ^19^F NMR signal by only a relatively small amount. This allowed obtaining good quality MRI images at a 20 mg/mL concentration of nanoparticles. Furthermore, the Gd(III) chelates shortened the T1 relaxation time of the bulk water protons and under optimized acquisition conditions, the quality of ^1^H MRI images was comparable to that obtained by using hydrogenated nanoparticles carrying Gd(III) chelates at the outermost surface of the monolayer. Although not yet optimal, the performance scored in a preclinical setup for **NP-C6OF-PEG/C6OF-DO3AGd** represents a valuable proof of principle for the design of contrast agents for dual ^1^H/^19^F MRI based on gold nanoparticles. The observation that Gd(III) units deeply embedded in the monolayer of water-soluble gold nanoparticles exert a reduced effect on the relaxation times of bulk water gives hints for an improved design of nanoparticle-based contrast agents. Our group is busily working in order to improve the ^19^F MRI properties of the NPs by increasing the number of fluorine nuclei per NP, and to develop second generation Gd(III) fluorinated chelates to find the best compromise for optimal ^1^H relaxivity and an acceptably short ^19^F T1.

## Figures and Tables

**Table 1:** T1 and T2 relaxation times determined on 40 mg/mL aqueous solutions of nanoparticles **NP-C8TEG/C8-DO3AGd-a** and **NP-C8TEG/C8-DO3AGd-b** and the respective calculated relaxivities.

Sample	T1 (ms)	T2 (ms)	r1 (s^−1^ mM^−1^)	r2 (s^−1^ mM^−1^)
NP-C8TEG/C8-DO3AGd-a	33.55 ± 0.04	6.43 ± 0.07	3.2	16.9
NP-C8TEG/C8-DO3AGd-b	14.49 ± 0.06	3.17 ± 0.06	4.9	22.5

**Table 2:** ^19^F Relaxation times determined for the fluorinated gadolinium-complexing ligand and **NP-C6OF-PEG/C6OF-DO3AGd** considering the signals at −90.1 ppm.

Sample	T1 ^a^ (ms)	T2 ^a^ (ms)	T1 ^b^ (ms)
HS-C6OF-DO3A	627	157	-
NP-C6OF-PEG	719	165	409 ± 24
NP-C6OF-PEG/C6OF-DO3AGd	258	33	233 ± 9

**^a^**: Data obtained at 470 MHz (11.75 T in CD_3_OD) using a nanoparticle concentration of 23.8 mg/mL corresponding to a Gd(III) chelate units of 3.04 mM; **^b^**: data obtained at 7 T on a 20 mg/mL solution of **NP-C6OF-PEG/C6OF-DO3AGd**, this corresponds to a concentration of 2.56 mM in Gd(III) chelate units.

## Supplementary Materials

The following are available online at https://www.mdpi.com/2079-4991/9/6/879/s1, Syntheses and characterization of ligands and nanoparticles used in this work and details of the acquisition sequences for relaxometric and MRI experiments.
