# Supplementary material for: Functionalized Gold Nanoparticles as Contrast Agents for Proton and Dual Proton/Fluorine MRI
Source: Nanomaterials (Basel). 2019 Jun 13;9(6):879. doi: 10.3390/nano9060879 (PMC6631171; doi:10.3390/nano9060879)
Supplement: Supplementary file 1 [file nanomaterials-09-00879-s001.pdf]

Functionalized Gold Nanoparticles as Contrast agents for Proton and dual Proton/Fluorine MRI

Maria Şologan 1,*, Francesco Padelli 2,*, Isabella Giachetti 2, Domenico Aquino 2, Mariangela Boccalon 1, Gianpiero Adami 1,3, Paolo Pengo 1 and Lucia Pasquato 1,3,*

[1] Department of Chemical and Pharmaceutical Sciences, University of Trieste, Via L. Giorgieri 1, 34127 Trieste, Italy; Mariangela.Boccalon@bracco.com (M.B.); gadami@units.it (G.A.); ppengo@units.it (P.P.)

[2] Neuroradiology Unit, Fondazione IRCCS Istituto Neurologico Carlo Besta, 20133 Milano, Italy; isabella.giachetti@istituto-besta.it (I.G.); Domenico.Aquino@istituto-besta.it (D.A.)

[3] INSTM Trieste Research Unit, Via L. Giorgieri 1, 34127 Trieste, Italy

* Correspondence: msologan@units.it (M.S.); francesco.padelli@istituto-besta.it (F.P.); lpasquato@units.it (L.P.); Tel.: +39-040-558-2406 (L.P.); +39-02-2394-3572 (F.P.)

Supplementary Material

Table of Content

1. General Information	Page S2
2. Synthesis of *N*-1-{2-[2-(2-Methoxyethoxy)ethoxy]ethyl}-8-sulfanyloctanamide, **HS-C8TEG**	Page S3
3. Synthesis of **NP-C8TEG**	Page S3
4. Synthesis of **HS-C8-DO3A**	Page S4
4.1 2-(8-bromo-octyl)-[1,3]dithiane-2-carboxylic acid ethyl ester, **A**	Page S5
4.2 10-bromo-2-oxo-decanoic acid ethyl ester, **B**	Page S5
4.3 10-bromo-2-hydroxy-decanoic acid ethyl ester, **C**	Page S6
4.4 2-Hydroxy-10-tritylsulfanyl-decanoic acid ethyl ester, **D**	Page S6
4.5 Synthesis of 2-methanesulfonyl-10-tritylsulfanyl-decanoic acid ethyl ester, **E**	Page S7
4.6 Coupling of the **DO3A^{t}Bu** with **E**	Page S7
4.7 Synthesis of thiol **HS-C8-DO3A**	Page S8
4.8 Synthesis of **HS-C8-DO3AGd**	Page S8
5. Synthesis of **HS-C6OF-PEG**	Page S9
5.1 6-Tritylthio-1-hexanol, **G**	Page S9
5.2 6-Tritylthio-1-(4-toluenesulfonyl)hexane, **H**	Page S10
5.3 Coupling of **H** with oxy-fluorinated tetraethylene glycol, giving the compound **I**	Page S10
5.4 Synthesis of TsOPEGOMe, **K**	Page S11
5.5 Coupling of **I** with **K**, giving the compound **L**	Page S11
5.6 Synthesis of thiol **HS-C6OF-PEG**	Page S12
6. Synthesis of **NP-C6OF-PEG**	Page S12
7. Synthesis of **HS-C6OF-DO3AGd**	Page S13
7.1 Synthesis of **TrtS-C6OF-OMs**, **M**	Page S13
7.2 Synthesis of **TrtS-C6OF-DO3A^{t}Bu**, **N**	Page S14
7.3 Synthesis of **HS-C6OF-DO3A**	Page S14
7.4 Synthesis of **HS-C6OF-DO3AGd**	Page S15
8. Characterization by magnetic resonance in a preclinical setup	Page S15
8.1 ^{1}H relaxometry and MRI Parameters	Page S15
8.2 ^{19}F T1 relaxometry and MRI parameters	Page S16
9. References	Page S17

1. General Information

All commercially available reagents were from Aldrich and Alfa Aesar, and used without purification unless otherwise mentioned. Solvents were purchased from Aldrich and VWR, deuterated solvents from Cambridge Isotope Laboratories and Aldrich. Dry solvents were obtained from Aldrich and Alfa Aesar. Chlorinated solvents were kept over K_2CO_3 with occasional shaking for at least 24 h prior to use. All other solvents were reagent grade and used as received. Reactions were monitored by TLC on Merck silica gel plates (0.25 mm) and visualized by UV light, I_2, or by $KMnO_4$–H_2SO_4. Chromatography was performed on Merck silica gel 60F-254 (230–400 mesh).

Nuclear Magnetic Resonance spectra were recorded on a Varian 500 spectrometer (operating at 500 MHz for proton, at 125 MHz for carbon, 470 MHz for ^{19}F), or on a Varian 400 MHz (operating at 400 for proton). ^{1}H NMR spectra were referenced to the residual protons in the deuterated solvent. ^{13}C NMR spectra were referenced to the solvent chemical shift. ^{19}F spectra were referenced to $CFCl_3$. Chemical shifts (δ) are quoted in ppm and the multiplicity of each signal is designated by the conventional abbreviations: s, singlet; d, doublet; t, triplet; q, quartet; m, multiplet; br, broad; dd, doublet of doublets. Coupling constants (J) are quoted in Hz.

Mass spectrometry measurements were obtained by electrospray ionization (ESI) with a Perkin Elmer APII at 5600 eV and recorded by Dr. Fabio Hollan, Department of Chemical and Pharmaceutical Sciences, University of Trieste, Italy.

UV-Visible spectroscopy measurements were recorded on a Shimadzu UV-1800 spectrophotometer.

Thermogravimetric analysis (TGA) were performed on TGA Q-500 V6.3 Build 189 using platinum pans and a heating rate of 10 °C/min up to 1000 °C or on a Netzsch STA 409 using alumina crucibles and a heating rate of 10 °C/min up to 650 °C under a static air atmosphere.

Transmission electron microscopy (TEM) images were obtained with either a Philips EM 208 operating at 100 kV or with a Joel 3010 high resolution electron microscope (1.7 nm point-to-point) operating at 300 keV using a *Gatan* slow-scan CCD camera (mod. 794).

Dynamic light scattering (DLS) measurements were performed on a Malvern Zetasizer Nano in backscatter mode. Analyses were performed on nanoparticle solutions with concentration of 10 mg/mL or 3.7 mg/mL, using either disposable micro cuvettes or disposable zeta cells (Malvern). The cell positioning factor was set to 0.83 mm and attenuator values of 5 or 9 were auto-optimized as a function of nanoparticles concentration.

ICP-OES: Total Au and Gd concentrations in the samples, solubilized with 1 mL of aqua regia (mixture HCl:HNO_3 of 3:1) and diluted to 10 mL with MilliQ water, were quantified by means of Inductively Coupled Plasma-Optical Emission Spectrometry (ICP-OES) using an Optima 8000 instrument (PerkinElmer; USA) equipped with an S10 integrated autosampler (PerkinElmer; USA).

The measurements were conducted using a calibration curve obtained by dilution of gold and gadolinium standard solutions for ICP–OES analysis (Sigma–Aldrich, Italy). The limit of detection in the obtained solutions at the operative wavelengths (Au 267.595 nm and Gd 376.839 nm) was 0.02 mg L^{-1} for both Au and Gd. The precision of the measurements expressed as repeatability (as RSD %) for the analysis was always less than 5%.

2. Synthesis of *N*-1-{2-[2-(2-Methoxyethoxy)ethoxy]ethyl}-8-sulfanyloctanamide, HS-C8TEG [1]

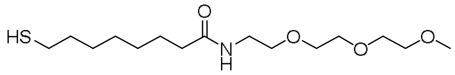

In a three neck round bottomed flask were dissolved 0.345 (0.169 mmol) of 7-({2-[2-(2-methoxyethoxy) ethoxy]ethyl}carbamoyl)heptyl ethanethioate in 15 mL of deoxygenated EtOH. At this solution was added solid $NaBH_4$ (0.53 g, 0.014 mol) and the mixture was allowed to stir for 1 hour at room temperature. The solution was acidified to pH 5 by adding HCl 1M. The mixture was diluted with 10 mL of AcOEt and 20 mL of water. The aqueous phase was extracted with AcOEt (3 x 15 mL) and the organic phases were washed with water (1 x 20 mL), dried over Na_2SO_4 and the solvent was evaporated under reduced pressure. The product was recovered under argon. Yield: 90%
^{1}H-NMR (500 MHz, $CDCl_3$) δ: 6.09 (br, 1H, N<u>H</u>); 3.57-3.68 (m, 10H, (OC<u>H</u>$_2$)$_4$); 3.47 (m, 2H, C<u>H</u>$_2$N); 3.40 (s, 3H, OC<u>H</u>$_3$); 2.52 (m, 2H, C<u>H</u>$_2$S); 2.19 (t, 2H, *J* =7.23, C<u>H</u>$_2$CO); 1.55-1.75 (m, 4H, C<u>H</u>$_2$); 1.28 - 1.40 (m, 6H, C<u>H</u>$_2$).

3. Synthesis of NP-C8TEG[1]

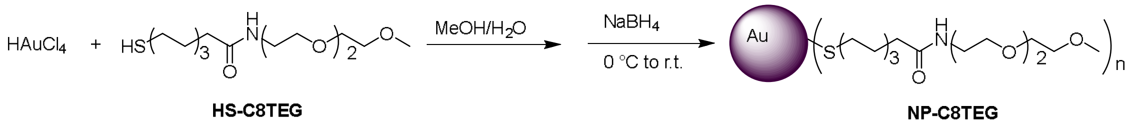

Scheme S1: Synthesis of **NP-C8TEG.**

To a solution of $HAuCl_4$ (0.0807 mg, 0.205mmol) in 40.35 mL of milliQ water was added a solution of **HS-C8-TEG** (0.132 g, 0.410 mmol) in 42.1 mL MeOH. The mixture was allowed to stir for 30 minutes and after this time, the solution was cooled at 0 °C and kept at this temperature for 30 min. To this mixture, a solution of $NaBH_4$ (0.081 g, 21 mmol) in 5.65 mL of milliQ water was added in 10 seconds and the reaction mixture was allowed to stir for 30 minutes at 0 °C and for 3 hours at room temperature. The solvent was removed and the solid residue washed with diethyl ether (5 x 30 mL),

the nanoparticles were transferred in two centrifuge tubes and washed with diethyl ether (5 x 30 mL). The nanoparticles purification was completed by size exclusion chromatography on Sephadex-LH20 using methanol as eluent. Solubility properties: Good solubility in water, methanol and DCM. ^{1}H-NMR (CD_3OD, 400 MHz) δ: 1.33 (br, CH_2), 1.65 (br CH_2), 2.18 (br, CH_2CO), 3.40 (br, OCH_3), 3.55 (br, CH_2N), 3.64 (br, OCH_2), 6.09 (br, 1H, NH). TEM: D = 1.7 nm; σ = 0.3 nm; n = 255. Hydrodynamic diameter (water): 8.3 ± 1.5 nm. TGA 31.4%. Average composition: Au_{220}C8TEG_{63}.

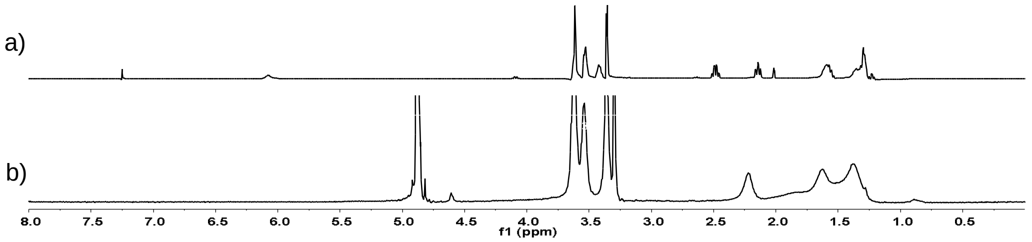

Figure S1: a) ^{1}H NMR (400 MHz, $CDCl_3$) of **HS-C8TEG** and b) ^{1}H NMR (400 MHz, CD_3OD) of **NP-C8TEG.**

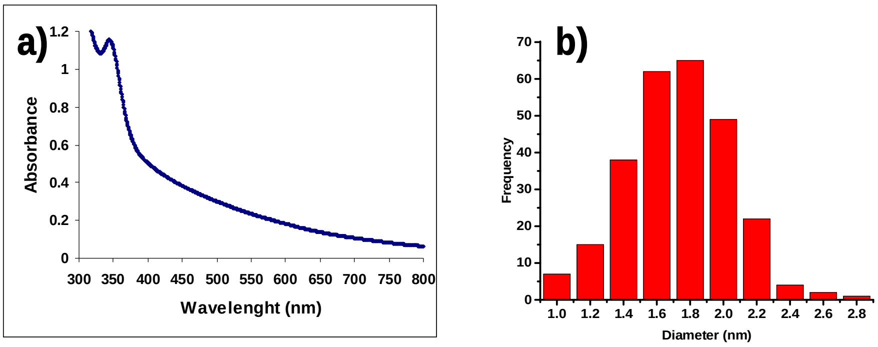

Figure S2: a) UV-VIS (0.1 mg/mL, $CHCl_3$) spectrum of **NP-C8TEG** and b) Size histogram of **NP-C8TEG.**

4. Synthesis of HS-C8-DO3A

The synthetic strategy for the preparation of the ligand **HS-C8-DO3A** is summarized in Scheme S2.

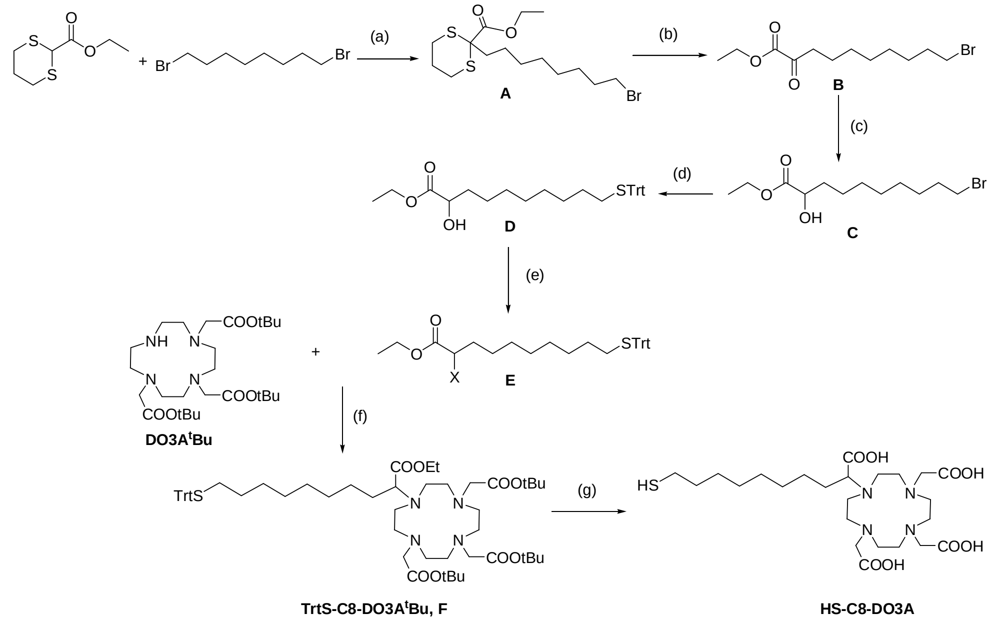

Scheme S2: Strategy for the synthesis of **HS-C8-DO3A**: (a) NaH, DMF; (b) NBS, DMF/H_2O (c) $NaBH_4$, MeOH (d) NaH, TrtSH, DMF (e) MsCl, Et_3N; (f) K_2CO_3, DCM; (g) 1. NaOH; 2. TFA, TIPS, DCM.

4.1 2-(8-bromo-octyl)-[1,3]dithiane-2-carboxylic acid ethyl ester, A[2]

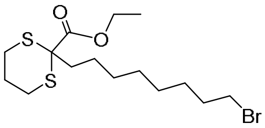

Working at 0 °C, a solution of 1,8-dibromooctane (3.5 mL, 19.03 mmol) and ethyl 1,3-dithiane-2-carboxylate (1.5 mL, 9.51 mmol) in dry DMF (3.5 mL) was added over 1 hour to a well stirred suspension of NaH (60% dispersion in mineral oil, 0.486 g, 10.46 mmol) in anhydrous toluene (10 mL). The suspension was stirred at room temperature overnight, then transferred into a separating funnel, diluted with 40 mL of diethyl ether and washed with water (2 x 40 mL). The organic solution was dried over Na_2SO_4 and the solvent evaporated *in vacuo* to give a yellow oil. The product was purified by flash chromatography on a silica gel column (EP to EP/AcOEt = 96/4). ^{1}H-NMR (500 MHz, $CDCl_3$) δ: 4.25 (q, 2H, J = 7.1, $\underline{CH_2}$-COO), 3.27 (t, 2H, J = 6.9, $\underline{CH_2}$-Br), 3.27 (t, 2H, J = 13.4, $\underline{CH_2}$-C), 2.6 (m, 2H, CH_2-CH_2), 2.05 (m, 2H, CH_2-CH_2), 1.7-2.0 (m, 4H, $\underline{CH_2}$-CH_2-Br, $\underline{CH_2}$-CH_2-C). ^{13}C-NMR (500 MHz, $CDCl_3$), 1.2-1.4 (m, 11 H, CH_2). Yield: 55%.

4.2 10-bromo-2-oxo-decanoic acid ethyl ester, B

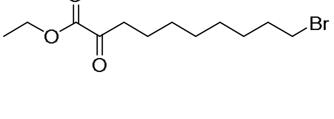

The crude 2-(8-bromo-octyl)-[1,3]dithiane-2-carboxylic acid ethyl ester (1.9 g, 4.95 mmol) was dissolved in acetonitrile (1 mL) and added over 1,5 hours to a well stirred suspension of NBS (7.06 g, 39.6 mmol) in 1.3 mL of acetonitrile and 2.4 mL water. After stirring for 2.5 hours at 0 °C, the red solution was poured into ice-cold DCM-hexane 1/1.4 (48 mL) and extracted with cold saturated $NaHSO_3$ (2 x 60 mL) and water (50 mL). The nearly colorless solution was cautiously washed with cold 20% Na_2CO_3 (1 x 30 mL), water (1 x 30 mL) and dried over Na_2SO_4. The product was obtained with a chemical yield of 90%.

^{1}H-NMR (500 MHz, $CDCl_3$) δ: 4.28 (q, 2H, *J* = 7.2, CH_2-COO), 3.40 (t, 2H, *J* = 6.8, CH_2-Br), 2.80 (t, 2H, *J* = 7.3, CH_2-CO), 1.8 (m, 2H, CH_2-CH_2-Br), 1.6 (m, 2H, CH_2-CH_2-CO), 1.2-1.4 (m, 21 H, CH_2). ^{13}C-NMR (500 MHz, $CDCl_3$) δ: 195.00 (CO), 162,00 (COO), 62.30 (CH_2-COO), 39.20 (CH_2-CO), 33.90 (CH_2-Br), 32.7 (CH_2-CH_2-Br), 28.03-29.05 (CH_2), 22.80 (CH_3-CH_2-COO).

4.3 10-bromo-2-hydroxy-decanoic acid ethyl ester, C

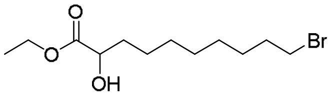

To a solution of 10-bromo-2-oxo-decanoic acid ethyl ester (1.2 g, 4 mmol) in 15 mL of MeOH solid $NaBH_4$ was added under stirring at 0 °C. After 30 minutes, the solvent was removed then the residue was taken-up with DCM (10 mL) and the resulting solution was washed with water (1 x 5 mL). The organic phase was dried over anhydrous sodium sulfate and the solvent was evaporated affording the product as yellowish oil with 85% yield.

^{1}H-NMR (500 MHz, $CDCl_3$) δ: 4.23 (q, 2H, *J* = 7.3, CH_2-COO), 4.14 (dd, 1H, *J* = 7.4 e 4.2, CH-OH), 3.40 (t, 2H, *J* = 6.9, CH_2-Br), 1.80 (m, 2H, CH_2-CH_2-Br), 1.75, 1.62 (m, 4H CH_2-CH-OH), 1.2-1.4 (m, 21 H, CH_2). ^{13}C-NMR (500 MHz, $CDCl_3$) δ: 175.37 (CO), 70.34 (CH), 61.64 (CH_2-COO), 34.30 (CH_2-CH), 33.98(CH_2-Br), 28.07-29.14 (CH_2), 24.65 (CH_3-CH_2-COO).

4.4 2-Hydroxy-10-tritylsulfanyl-decanoic acid ethyl ester, D[3]

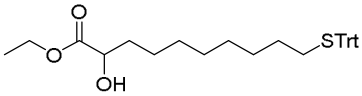

To a suspension of NaH (60% dispersion oil, 0.15 g, 3.73 mmol) in dry DMF (3.2 mL) maintained at 0 °C, was added TrtSH (0.939 g, 3.4 mmol). The mixture was allowed to stir for 30 minutes and afterwards a solution of compound **C** (1 g, 3.4 mmol) in 3.2 mL dry DMF was added. The mixture was left stirring overnight at room temperature and after that a mixture of EP/Ethyl ether = 4/1 was added to precipitate the NaBr. The solid was filtered off and the solution was brought to dryness, the

crude was purified by flash chromatography using silica gel, solvent EP/AcOEt = 98/2 giving a yellow oil with a 70 % yield.

^{1}H-NMR (500 MHz, $CDCl_3$) δ: 7.2-7.4 (m, 15H, STrt), 4.23 (q, 2H, *J* = 7.2, <u>CH_2</u>-COO), 4.14 (dd, 1H, *J* = 7.5, 4.2, CH-OH), 2.14 (t, 2H, *J* = 7.2, <u>CH_2</u>-STrt), 1.60, 1.75 (m, 4H, <u>CH_2</u>-CH), 1.15-1.38 (m, 21 H, CH_2). ^{13}C-NMR (500 MHz, $CDCl_3$) δ: 175.38 (CO), 145.04 (<u>STrt</u>), 126.46, 127.75, 129.57 (STrt) 70.40 (CH), 61.58 (CH_2-COO), 34.36 (CH_2-CH), 31.99 (<u>CH_2</u>-STrt), 28.55-28.92 (CH_2), 24.66 (CH_3-CH_2-COO).

4.5 2-methanesulfonyl-10-tritylsulfanyl-decanoic acid ethyl ester, E[4]

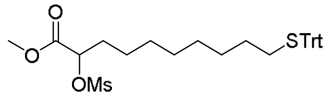

A solution of 2-hydroxy-10-tritylsulfanyl-decanoic acid ethyl ester, **D** (0.135 g, 0.275 mmol) in 1 mL of acetonitrile was brought to 0 °C and triethylamine (80 µL, 0.55 mmol) and MsCl (70 µL, 0.826 mmol) were added dropwise. After 3.5 hours other 0.5 mL of acetonitrile, 40 µL Et_3N and 70 µL MsCl were added. After 7 hours the reaction was complete and the mixture was washed with saturated NH_4Cl (1 x 20 mL) and the organic phase was washed with brine (1 x 20 mL). The solvent was removed *in vacuo* and the product was purified by flash chromatography using silica gel, and eluting with EP/AcOEt = 98/2. The product was obtained in 30% yield.

^{1}H-NMR (500 MHz, $CDCl_3$) δ: 7.2-7.4 (15H, STrt), 4.98 (dd, 1H, *J* = 7.7 and 4.7, CH-OH), 4.23 (q, 2H, *J* = 7.2, <u>CH_2</u>-COO), 3.13 (s, 3H, CH_3), 2.15 (t, 2H, *J* = 7.3, <u>CH_2</u>-STrt), 1.86 (m, 2H, CH_2-CH), 1.15-1.38 (m, 21H, CH_2).

4.6 Coupling of the DO3A^{t}Bu with 2-methanesulfonyl-10-tritylsulfanyl-decanoic acid ethyl ester, F[5]

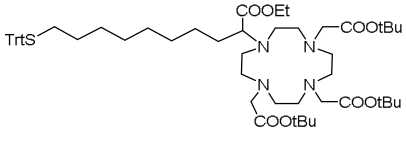

DO3A^{t}Bu (0.695 g) was added to a suspension of K_2CO_3 (0.466g, 3 equiv) in 8 mL acetonitrile and let to stir at 52 °C. At this mixture a solution of 2-methanesulfonyl-10-tritylsulfanyl-decanoic acid ethyl ester, **E** (0.62g, 1 equiv) in 3 mL of acetonitrile was added in 1 hour. The reaction mixture was left stirring overnight at 52 °C. The solvent was removed and the crude product was purified using a flash chromatography using DCM to DCM/MeOH = 98/2 as eluent. The compound **K** was obtained with a 52% yield.

^{1}H-NMR (500 MHz, $CDCl_3$) δ: 7.2-7.4 ppm (m, 15H, STrt), 4.23 (q, 2H, *J* = 7.2, $\underline{CH_2}$-COO), 2.3-3.2 (br, 16 H, CH_2N), 3.13 (s, 3H, CH_3), 2.15 (t, 2H, *J* = 7.3, $\underline{CH_2}$-STrt), 1.10-1.6 ppm (m, 21H, CH_2, CH_3). ^{13}C-NMR (500 MHz, $CDCl_3$) δ 172.9 (CO), 145.05 (Trt), 129.56, 127.77,126.49 (Trt), 61.49 (CH-COO), 49.00-54.95 ppm (CH_2-COO), 29.62-30.03 ppm (CH_2), 29.02 ($\underline{CH_2}$-STrt). ESI (MeOH) m/z: 1009 [M + Na^+], 987 [M + H^+].

4.7 Synthesis of thiol HS-C8-DO3A[6]

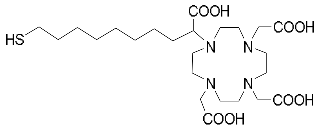

NaOH 5% (2.6 mL) was added to compound **TrtS-C8-DO3A^{t}Bu, F,** in 2.5 mL dioxane and the reaction was let to stir for one week adding in this period another 3 mL of NaOH 5%. Then, HCl 10 % was added and the solvent was evaporated. The solid was dissolved in 2 mL of HCl 1M and then extracted with AcOEt/MeOH 1/1 (6 mL). The organic phase was evaporated giving a yellow oil with a 15% yield. ^{1}H-NMR (500 MHz, $CDCl_3$) δ: 7.2-7.4 ppm (m, 15H, STrt), 2.3-3.2 (br, 16 H, CH_2N), 3.13 (s, 3H, CH_3), 2.15 (t, 2H, *J* = 7.3, $\underline{CH_2}$-STrt), 1.10-1.6 ppm (m, 18H, CH_2, CH_3). ^{13}C-NMR (500 MHz, $CDCl_3$) δ 172.9 (CO), 145.05 (Trt), 129.56, 127.77,126.49 (Trt), 61.49 (CH-COO), 29.62–30.03 ppm (CH_2), 29.02 ($\underline{CH_2}$-STrt).

TFA (0.8 mL) was added to this compound (0.05 g, 0.052 mmol) and the solution became deep yellow, the mixture was diluted with 0.5 mL deoxygenated DCM and the reaction was let to stir overnight. Then the solvent was evaporated and to the yellowish solid was added TFA (0.2 mL) (the solution became yellow), TIPS (0.2 mL) (the solution became almost colorless) and deoxygenated DCM (1.5 mL). The reaction was allowed to stir for 2 hours and the solvent was removed *in vacuo.* Then the solid was washed five times with hexane, after removal of trace of hexane, a white solid was obtained. In the ^{1}H –NMR spectra, some small peaks at about 7.1 ppm could be seen and were assigned to the residual presence of triphenylmethane.

^{1}H-NMR (500 MHz, CD_3OD) δ: 2.6-3.7 (br, 16 H, CH_2N), 2.51 (t, 2H, *J* = 7.3, $\underline{CH_2}$-SH), 1.10-1.6 ppm (m, 2H, CH_2). ^{13}C-NMR (500 MHz, CD_3OD) δ 162.72 (CO), 61.49 (CH-COO), 49.00-54.95 ppm (CH_2-COO), 29.62–30.03 ppm (CH_2), 29.02 ($\underline{CH_2}$-SH).

4.8 Synthesis of HS-C8-DO3AGd[6]

20 mg of **HS-C8-DO3A** (0.036 mmol) were dissolved in 1.5 mL of deoxygenated HEPES (pH 7.4, 0.1 M in H_2O mQ). At this solution 0.330 mL of a 0.1 M $GdCl_3$ solution in water (0.033 mmol, 0.9

equiv.) were added and the reaction was allowed to stir at 25 °C for 2 h. The xylenol orange test suggests the complete complexation of the Gd(III). This solution was used for the synthesis of mixed monolayers without further treatments.

5. Synthesis of HS-C6OF-PEG[7]

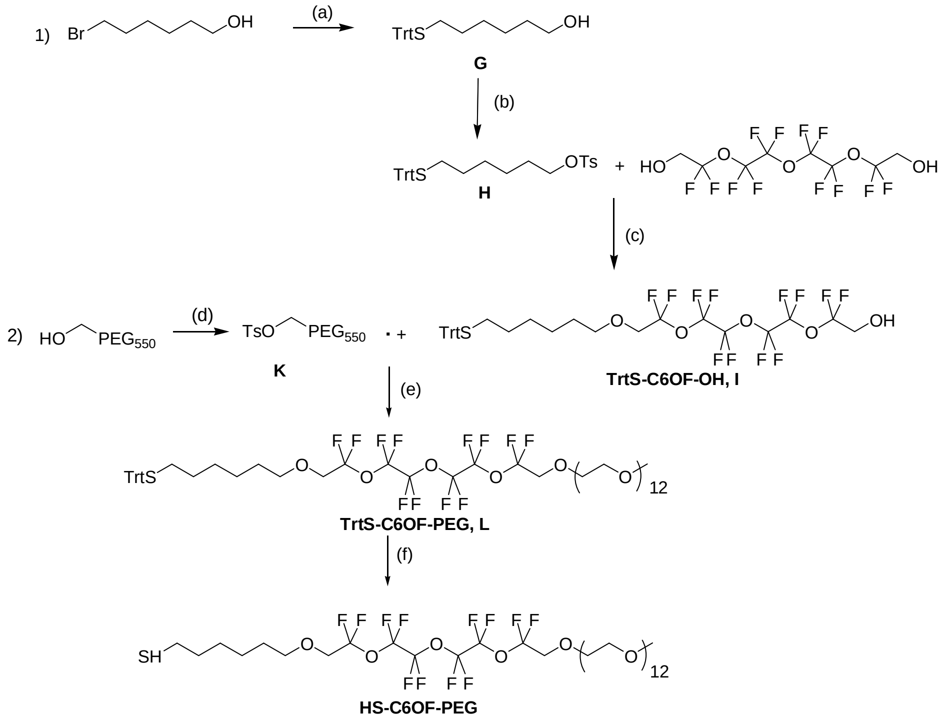

Scheme S3: Synthesis of thiol **HS-C6OF-PEG**: (a) TrtSH, K_2CO_3, EtOH/H_2O = 1/1, 90 °C, overnight; (b) TsCl, Et_3N, DCM, r.t., overnight; (c) KOH, dioxane, reflux, overnight; (d) TsCl, DCM, r.t., overnight; (e) NaH dioxane, 50 ^{0}C, overnight; (f) TFA, TIPS, DCM, r.t. 4 h .

5.1 6-Tritylthio-1-hexanol, G

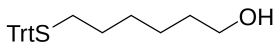

1-Bromo-hexanol (2.5 mL, 19.1 mmol), tritylthiol (5.28 g, 19.1 mmol) and potassium carbonate (5.28 g, 38.2 mmol) were dissolved in 200 mL of H_2O/EtOH 1:1 mixture forming a suspension. The reaction mixture was refluxed at 90 ^{o}C for 18 hours. After the reaction was complete, the residue was neutralized with 1M HCl (150 mL) and extracted with DCM (5 x 100 mL). The organic layers were collected and washed with water (3 x 150 mL) and dried over Na_2SO_4. A yellow solid was obtained in quantitative yield.

^{1}H-NMR (400 MHz, $CDCl_3$) δ: 7.4-7.40 (m, 6H, Trt),7.30-7.25 (m, 6H, Trt), 7.22-7.18 (m, 3H, Trt), 3.57 (t, 2H, J = 6.6, C$\underline{H_2}$OH); 2.14 (t, 2H, J = 7.3, CH_2STrt), 1.55-1.40 (m, 4H, CH_2), 1.27-1.22 (m, 4H, CH_2). ^{13}C-NMR (67.8 MHz, $CDCl_3$) δ: 145.15 (C1 Trt), 129.68 (C3 Trt), 127.88 (C2 Trt), 126.59

(C4 Trt), 66.36 (C_q), 62.80 (CH_2OH), 32.39 (CH_2), 31.78 (CH_2), 28.60 (CH_2), 28.41 (CH_2), 25.15 (CH_2).

5.2 6-Tritylthio-1-paratoluenesulfonylhexane, H

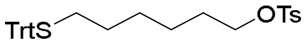

The *p*-toluenesulfonyl chloride (TsCl) (789 mg, 4.14 mmol) was dissolved under argon atmosphere in 3 mL of dry DCM. The mixture was cooled at 0 °C and added drop wise to a mixture of 6-tritylthio-1-hexanol, **G** (1.5 g, 3.98 mmol), triethylammine (1.11 mL, 7.96 mmol) in 1 mL of dry DCM. After two hours the reaction mixture was diluted with DCM (30 mL) and water (30 mL). The aqueous layer was extracted with DCM (4 x 30 mL). The organic layers were collected and washed with HCl 6 N (1 x 30 mL), $NaHCO_3$ 5% (2 x 40 mL) and H_2O (2 x 40 mL), dried over Na_2SO_4, filtered and the solvent removed under reduced pressure. The product was purified by flash chromatography, using $CHCl_3$/Petroleum ether 6/4 as eluent obtaining a white solid. Yield: 92%.

^{1}H-NMR (270 MHz, $CDCl_3$) δ: 7.79 (m, 2H, Ts), 7.42-7.21 (m, 17H, Trt+Ts), 3.98 (m, 2H, CH_2OTs); 2.44 (s, 3H, CH_3), 2.11 (t, 2H, J = 7.3, CH_2STrt), 1.53 (m, 2H, CH_2), 1.28 (m, 2H, CH_2), 1.18 (m, 2H, CH_2) ppm. ^{13}C-NMR (67.8 MHz, $CDCl_3$) δ: 145.15, 144.77, 133.30, 129.9, 127.9, 126.64, 70.41, 66.42, 31.63, 28.47, 24.78, 22.54, 21.51, 13.99 ppm. MS-ESI (m/z): 553.2 [$M+Na^+$], 569.2 [$M+K^+$].

5.3 Coupling of H with oxy-fluorinated tetraethylene glycol, giving the compound I

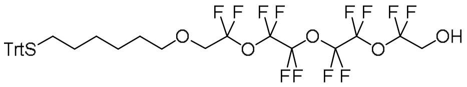

To a solution of perfluorinated tetraethylene glycol (1.383g, 3.382 mmol) in 2 mL of dry dioxane a solution of 6-tritylthio-1-paratoluenesulfonylhexane, **H** (0.816g, 1.537 mmol) in 2 mL of dry dioxane was added, the addition was completed in 10 minutes. To the reaction mixture, KOH pellets (0.283 g, 5.07 mmol) were added and the reaction mixture was stirred overnight at 100 ^{o}C. After the reaction was complete, the residue was dissolved in 30 mL of water and the mixture was extracted with ethyl acetate (5 x 20 mL). The organic phase was then washed with water (2 x 25 mL) and brine (2 x 25 mL) and dried over Na_2SO_4, filtered and evaporated under reduced pressure. The product was purified by flash chromatography using hexane/AcOEt 9/1 as eluent. Yield 56%.

^{1}H-NMR (400 MHz, $CDCl_3$) δ: 7.43 (m, 6H, Trt), 7.28 (m, 6H, Trt), 7.26 (m, 3H, Trt), 3.89 (t, 2H, J = 9.6, HOC$\underline{H}_2$CF$_2$); 3.78 (t, 2H, J = 9.7, OC$\underline{H}_2$CF$_2$); 3.55 (t, 2H, J = 6.5, OC$\underline{H}_2$CH$_2$); 2.15 (t, 2H, J =

7.3, CH_2STrt), 1.50 (m, 2H, CH_2), 1.28 (m, 2H, CH_2), 1.18 (m, 4H, CH_2). MS-ESI (m/z): 791.3 [$M+Na^+$].

5.4 TsOPEGOMe, K

A solution of TsCl (789 mg, 4.14 mmol) in 3 mL dry DCM was added drop wise to a mixture of HO-PEG-OMe (2.01 mL, 3.98 mmol) and triethylammine (1.11 mL, 7.96 mmol) diluted with 1 mL dry DCM and kept at 0 °C. The mixture was allowed to stir at room temperature for 18 hours. The mixture was then diluted with in DCM (50 mL) and water (50 mL). The aqueous layer was extracted with DCM (3 x 50 mL). The organic layers were collected,washed with HCl (x mL), $NaHCO_3$ 5% (3 x 30 mL) and H_2O (3 x 30 mL) and dried over Na_2SO_4. After removal of the solvent under reduced pressure, the crude product was purified by flash chromatography using $CHCl_3$ as eluent. A white solid was obtained in 92% yield. ^{1}H-NMR (270 MHz, $CDCl_3$) δ: 7.81 (d, 2H, *J* = 8.1, Ts), 7.34 (d, 2H, *J* = 8.1, Ts), 4.15 (t, 2H, *J* = 4.7, CH_2OTs); 3.67-3.52 (m, 42H, OCH_2), 3.38 (s, 3H, OCH_3), 2.45 (s, 3H, CH_3).

5.5 Coupling of I with K giving the compound L

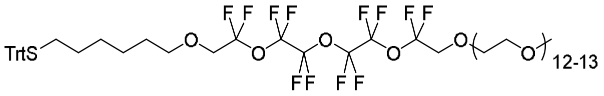

The alcohol **TrtS-C6OF-OH, I** (0.29 g, 0.377 mmol) was dissolved in 3 mL dry dioxane under Ar atmosphere. At the solution NaH (36.2 mg, 1.508 mmol, previously washed 5 times with petroleum ether) was added and the mixture was stirred for 20 minutes. To the solution **TsOPEGOMe, K** (0.266 g, 0.377 mmol) dissolved in 2 mL dry dioxane was added over a time period of 10 minutes, the solution became yellow. The reaction mixture was heated under stirring at 50 °C for 18 h, then it was cooled at room temperature and diluted with $CHCl_3$. The organic layer was washed with brine (3 x 20 mL) and dried over Na_2SO_4. After filtration and removal of the solvent, the product was purified by flash chromatography using $CHCl_3$/AcOEt 9/1 as eluent. Yield: 90%.

^{1}H-NMR (400 MHz, $CDCl_3$) δ: 7.44 (m, 6H, Trt), 7.25 (m, 6H, Trt), 7.23 (m, 3H, Trt), 3.96 (t, 2H, *J* = 9.9, $OC\underline{H}_2CF_2$); 3.6-3.5 (m, 4H, CH_2O + OCH_2CF_2); 3.36 (s, 3H, OCH_3); 2.13 (t, 2H, *J* = 7.3, CH_2STrt), 1.49 (m, 2H, CH_2), 1.38 (m, 2H, CH_2), 1.21 (m, 4H, CH_2).

5.6 Synthesis of thiol HS-C6OF-PEG

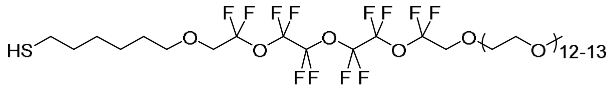

Compound **TrtS-C6OF-PEG, L** (518 mg, 0.399 mmol) was dissolved in deoxygenated dichloromethane (5 mL) under argon atmosphere. To the mixture, trifluoroacetic acid (0.59 mL, 7.98 mmol) and then triisopropyl silane (0.16 mL, 0.798 mmol) were added. The reaction mixture was stirred at room temperature for 4 hours and then the solvent was removed *in vacuo,* the residue was co-evaporated with methanol. The crude product was purified by column chromatography over silica gel using chloroform-ethyl acetate (9:1 v/v) as eluent. Compound **HS-C6OF-PEG** was obtained as a colorless oil with a 97% yield.

^{1}H-NMR (400 MHz, $CDCl_3$) δ: = 3.92 (t, 2H, *J* = 9.9, $OC\underline{H_2}CF_2$); 3.76 (m, 4 H, CH_2O + OCH_2CF_2), 3.67 (s, 3H, CH_2O); 3.39 (s, 3H, OCH_3), 2.15 (t, 2H, *J* = 7.3, CH_2SH), 1.49 (m, 2H, CH_2), 1.38 (m, 2H, CH_2), 1.21 (m, 4H, CH_2). ^{19}F NMR (470 MHz, CD_3OD) = -83.6 to -84.0 (m, CF_2), -94.6 to -95.12 (m, CF_2-O).

6. Synthesis of NP-C6OF-PEG

Tetrachloroauric acid (0.058 g, 0.1465 mmol) was dissolved in 50 mL of deoxygenated water, to this solution thiol **HS-C6OF-PEG** (0.310 g, 0.293 mmol) dissolved in 50 mL deoxygenated methanol was than added. The mixture was stirred for 30 minutes at room temperature and for 30 minutes at 0 ^oC. At 0 ^oC a freshly prepared solution of $NaBH_4$ (0.061 g, 1.6115 mmol) was added rapidly. Then the reaction mixture was stirred for 1 hour at 0 ^oC and 2 hours at room temperature. After this time the reaction was stopped and the precipitation of the nanoparticles was attempted without prior concentrating the solution using different solvent such as methanol, hexane, pentane, diethyl ether, acetone but no precipitation could be achieved. The ethanol was then removed without exceedingly concentrating the solution and adding milliQ water at small portions. The resulting solution was dialyzed for 3 days against water. Then the solvent was removed and the brown solid was washed two times with diethyl ether (2 x 15 mL). 0.080 g of **AuNPs-C6OF-PEG** were obtained. ^{1}H-NMR (CD_3OD, 400 MHz) δ: 3.38 (br, CH_3O), 3.48-3.77 (br, CH_2O), 4.10 (br, CF_2CH_2O). UV-VIS λ: monotonic decrease from 200 nm, absence of surface plasmon band. TEM: D = 1.5 nm; σ = 0.3 nm; n = 217. TGA: 68.5 %. Average composition: $Au_{130}C6OFPEG_{53}$.

7. Synthesis of HS-C6OF-DO3AGd,

This strategy for the preparation of the ligand **HS-C6OF-DO3AGd** is summarized in Scheme S4.

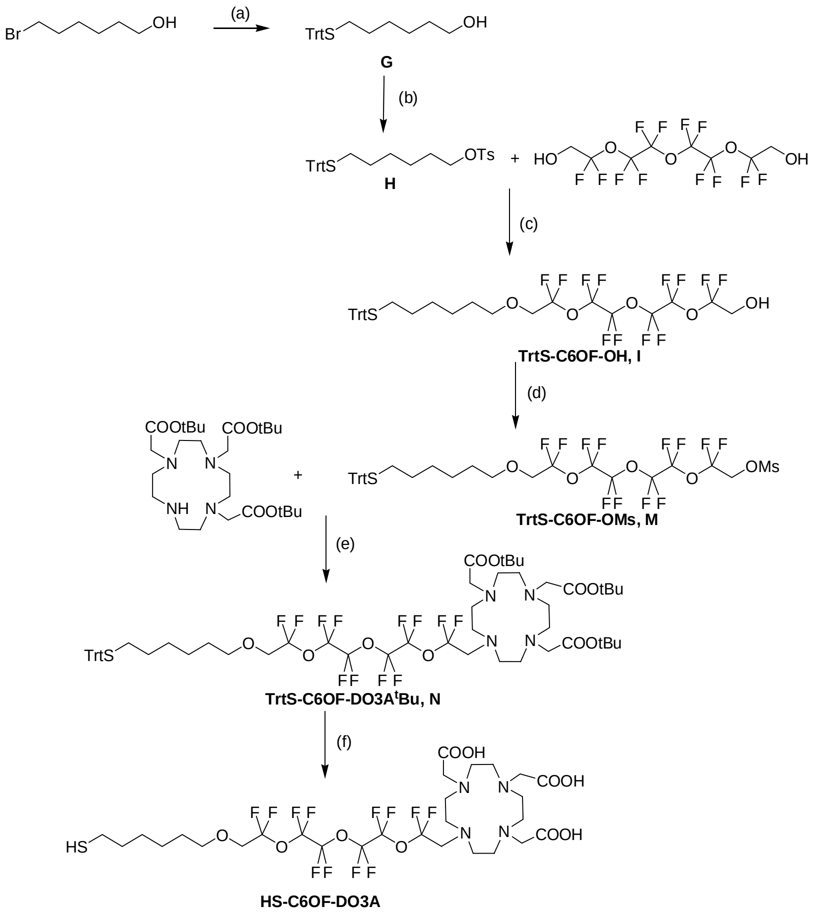

Scheme S4: Synthesis of **HS-C6OF-DO3A**: (a) TrtSH, K_2CO_3, EtOH/H_2O; (b) TsCl, Et_3N, DCM; (c) NaH, dioxane;(d) MsCl, Et_3N, acetonitrile; (e) K_2CO_3, acetonitrile; (f) TFA, TIPS.

7.1 TrtS-C6OF-OMs, M

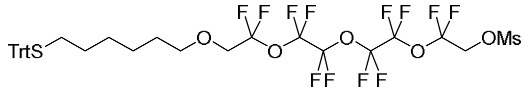

To a solution of **I** (0.195 g, 0.254 mmol) in 3.5 mL acetonitrile cooled at 0 °C Et_3N (0.113 mL, 0.812 mmol) and MsCl (0.127 mL, 1.653 mmol) were added dropwise. The reaction was let to stir for 3 hours under argon atmosphere. Then the solvent was removed and the crude mixture was purified by flash chromatography eluting with DCM. The pure product was used immediately for the next reaction.

7.2 TrtS-C6OF-DO3A^{t}Bu, N

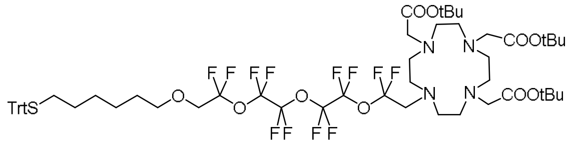

The **M** compound (0.170 g, 0.2 mmol) dissolved in 2 mL of acetonitrile was added over a time interval of 2 hours to a solution prepared by dissolving DO3A^{t}Bu (0.205 g, 0.4 mmol) in 2 mL of acetonitrile, containing K_2CO_3 (0.220 g, 1.6 mmol), and kept at 60 °C. The reaction mixture was left stirring overnight. Then 0.111 g of K_2CO_3 and 0.098 g of DO3A^{t}Bu were added and the mixture was stirred at 60 °C for additional 18 h. A further amount of 0.094 g of K_2CO_3 and 0.076 g of DO3AtBu was added and the reaction mixture was maintained at 60 °C for other 6 hours. After the last addition of reagents, the ^{1}H NMR spectrum demonstrated the disappearance of the signals corresponding to the compound **M**. Yield: 58%.

^{1}H-NMR (400 MHz, CD_3OD) δ: 7.43 (m, 6H, Trt), 7.28 (m, 6H, Trt), 7.26 (m, 3H, Trt), 3.82 - 3.88 (br, 2H, CH_2N), 3.78 (t, 2H, *J* = 10.3, $OC\underline{H}_2CF_2$), 3.53 (t, 2H, *J* = 6.5, $OC\underline{H}_2CH_2$); 3.32 (m, 2H, N-$\underline{CH_2}$-CF_2); 3.26 (br, 2H, $\underline{CH_2}$-COO); 2.95; 2.76 (br s, 2H, CH_2N), 2.13 (t, 2H, *J* = 7.3, CH_2STrt), 1.6-1.15 (m, CH_2, tBu). ^{13}C-NMR (67.8 MHz, CD_3OD) δ: 144.99 (CO), 129.60, 127.70, 126.50 (Trt), 47.00 – 46.70 ($\underline{CF_2}CH_2N$), 69.90, 69.64 (t, *J* = 29.9, $O\underline{CH_2}CF_2$), 72.70 ($O\underline{CH_2}CH_2$), 54.20 ($\underline{CH_2}N$), 58.40 ($\underline{CH_2}COO^tBu$), 55.35, 52.40 ($\underline{CH_2}N$)32.07 ($\underline{CH_2}STrt$), 28.16, 28.20, 28.10 ($\underline{C}CH_3$).

7.3 Synthesis of HS-C6OF-DO3A

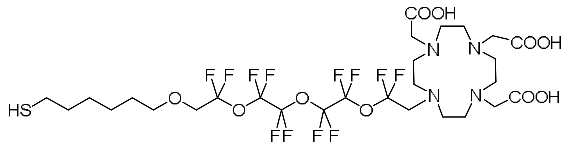

Trifluoroacetic acid (0.9 mL, 11.8 mmol) was added to a solution of **N** (0.075 g, 0.059 mmol) in 1 mL of anhydrous DCM; the reaction mixture was allowed to stir overnight. Then the solvent was removed and to the solid residue were added 2 mL anhydrous deoxygenated DCM, 0.28 mL TFA and 0.28 mL of TIPS. The reaction was left stirring for 2h, the solvent was removed and the solid was washed five times with deoxygenated hexane. The solid was dried under argon and the product was obtained as a white solid. Yield 100 %.

^{1}H-NMR (400 MHz, CD_3OD) δ: 3.8-3.92 (br, 2H, CH_2N), 3.79 (t, 2H, *J* = 10.3, $OC\underline{H}_2CF_2$), 3.55 (t, 2H, *J* = 6.5, $OC\underline{H}_2CH_2$); 3.04-2.66 (m, 16H, CH_2N), 2.53 (t, 2H, *J* = 7.3, CH_2-SH,); 1.50 (m, 2H,

CH_2); 1.28 (m, 2H, CH_2), 1.18 (m, 4H, CH_2). ^{19}F NMR (470 MHz, CD_3OD) δ: -76.9 (m, CF_2CH_2N), -81.6 to -80.9 (m, CF_2), -82.79 (m, CF_2), -94.11 to -93.6 (m, CF_2O).

7.4 Synthesis of HS-C6OF-DO3AGd

HS-C6OF-DO3A (0.045 g, 0.045 mmol) were dissolved in 2.5 mL of deoxygenated HEPES (pH 7.4, 0.1 M in H_2O mQ). To this solution 0.413 mL of a 0.1 M $GdCl_3$ solution (0.041 mmol, 0.9 equiv) were added and the reaction mixture was stirred for 2 h at 25 °C. This solution was used for the place exchange reaction without further purification.

8. Characterization by magnetic resonance in a preclinical setup

8.1 ^1H relaxometry and MRI Parameters

T1 and T2 relaxation times were assessed by a set of Rapid Acquisition with Relaxation Enhancement (RARE) sequences varying repetition time (TR) and echo time (TE) respectively. For T1 mapping, TE was set to 5.81 ms, while TR values were 50,60,70,80,90,100,120,130,140,150,200,250,300,350,400,450,500,800,1500,3000,5500, 7000,10000 ms.

In T2 mapping TR was set to 2000 ms, while TE ranged from 6.2 ms to 620 ms with 6.2 ms steps.
Mean signal estimated within an appropriate Region of Interest (ROI) was plotted with respect to different TR or TE values and then fitted with the following functions:

$$S = A\,[\,1 - \exp(-\,TR / T1)\,] + B$$

$$S = A \exp(-\,TE / T2) + B$$

where A is the maximum signal intensity and B is the image absolute bias.
For all of the samples sample ^1H MRI the parameters of the RARE sequence were setup according to the pertinent T1 and T2 values, Table 1 collects T1 weighted (T1-w), T2 weighted (T2-w) and spin density weighted (SD-w) sequences key parameters.

Table 1. ^{1}H MRI sequences parameters at 7 T.

	**NP-C8TEG/C8-DO3AGd-b**	**NP-C8TEG/C8-DO3AGd-a**	**NP-C6OF-PEG**		**NP-C6OF-PEG/C6OF-DO3AGd**	
	**T1-w**	**T2-w**	**T1-w**	**SD-w**	**T1-w**	**T2-w**
**TR [ms]**	5	500	500	6000	50	1600
**TE [ms]**	2.88	9	5.35	90	5.35	90
**Flip Angle**	90°<br>180°					
**Receiver BW [Hz]**	100 000	50 000	100 000	25 000	10 000	25 000
**Slice orientation**	Coronal					
**Slice thickness [mm]**	0.6					
**FOV [mm]**	16 x 16					
**Matrix**	160 x 160					
**Averages numbe**	5000	40	100	20	1200	50
**Acquisition time**	1h	1h	55 m	1h30m	1h06 m	1h

Due to its very short relaxation times, **NP-C8TEG/C8-DO3AGd-b** sample required short TR and TE values and therefore even receiver bandwidth and pulse duration had to be adjusted accordingly. On the other hand, because of its long T2 value, for **NP-C6OF-PEG** solution a T2-weighted imaging sequence was not feasible due to TE and TR too long values required. Therefore a spin density-weighted sequence was optimized for this sample.

8.2 ^{19}F T1 relaxometry and MRI parameters

Fluorine T1 relaxation time values were then estimated. The maps were obtained acquiring a multiple-TR RARE sequence (TR = 3000, 1000, 800, 600, 400, 200, 100 ms) of 20 mg/mL solutions of **NP-C6OF-PEG** and **NP-C6OF-PEG/C6OF-DO3AGd** samples.

The parameters for the acquisition of the ^{19}F MRI are reported in Table 2.

Table 2. ^{19}F MRI sequences parameters at 7 T.

	**NP-C6OF-PEG**	**NP-C6OF-PEG/C6OF-DO3AGd**
**TR [ms]**	2000	200
**TE [ms]**	6	3
**Flip Angle**	90°<br>180°	
**RARE factor**	8	4
**Excitation frequency [MHz]**	282.5602	282.5614
**Excitation BW [Hz]**	2 000	3 500
**Receiver BW [Hz]**	10 000	50 000
**Slice thickness [mm]**	5	
**FOV**	16 x 16	
**Matrix**	32 x 32	
**Averages number**	500	4000
**Acquisition time**	1 h 6 m	1 h 46 m

9. References

1. Pengo, P.; Polizzi, S.; Battagliarin, M.; Pasquato, L.; Scrimin, P. *J. Mater. Chem.* **2003**, 13, 2471.
2. Graham, D. W., Ashton, W.T., Barash, L., Brown, J.E., Brown, R. D., Canning, L.F., Chen, A., Springer, J.P., Rogers, E.F. *J. Med. Chem.* **1987,** 30, 1074.
3. Raghunand, N., Jagadish, B., Trouard, T.P., Galons, J.-P., Gillies, R. J., Mash, E.A. *Magn Reson. Med.* **2006**, 55, 1272.
4. Hurski, A. L., Kulinkovich, O. G. *Russ J. Org. Chem.* **2011**, 47, 1653.
5. Levy, S. G., Jacques, V., Zhou, K.L., Kalogeropoulus, S., Schumacher, K., Amedio, J. C., Scherer, J. E., Witovski, V. R.,Lombardi, R., Koppetsch, K. *Org. Process Res. Dev.* **2009**, 13, 535.
6. Irure, A.; Marradi, M.; Arnaiz, B.; Genicio, N.; Padro, D.; Penadez, S. *Biomater. Sci.* **2013**, 1, 658.
